# Application of Absolute Alcohol in the Treatment of Traumatic Intracranial Hemorrhage via Interventional Embolization of Middle Meningeal Artery

**DOI:** 10.3389/fneur.2020.00824

**Published:** 2020-08-06

**Authors:** Gangxian Fan, Henglu Wang, Jinke Ding, Chao Xu, Yongliang Liu, Chao Wang, Zefu Li

**Affiliations:** Department of Neurosurgery, Binzhou Medical University Hospital, Binzhou, China

**Keywords:** absolute alcohol, embolization, acute epidural hematoma, chronic subdural hematoma, middle meningeal artery

## Abstract

**Background:** Traumatic brain injury is a common condition in neurosurgery. Traditional methods of treatment include conservative treatment and surgical evacuation using burr-holes or craniotomy; however, studies have reported problems such as high re-expansion rates after conservative treatment of epidural hematoma and high postoperative recurrence rates of subdural hematoma. Solutions to these problems are lacking, and research into new treatment methods is ongoing. Among the potential new treatments, middle meningeal arterial embolization is an option. This study involved patients with acute epidural hematoma and chronic subdural hematoma. The purpose was to evaluate the use and effects of absolute alcohol to embolize the middle meningeal artery to treat intracranial hematoma.

**Material and Methods:** A retrospective description study was 12 cases of intracranial hematoma who treated with absolute alcohol interventional therapy from our hospital between June 2018 and October 2019. Five patients with acute epidural hematoma and seven patients with chronic subdural hematoma were treated using absolute alcohol to embolize the middle meningeal artery. Patients' clinical data, imaging results, surgical results, and prognosis were recorded and analyzed.

**Results:** All patients underwent absolute alcohol embolization of the middle meningeal artery, in combination with burr-hole drainage. All imaging data were confirmed preoperatively. We successfully used absolute alcohol to embolize the middle meningeal artery intraoperatively and confirmed these results by postoperative angiography. All patients achieved symptomatic relief without complications, and no recurrence or re-expansion was seen with follow-up computed tomography. Our study has been registered in the Chinese Clinical Trial Registry (http://www.chictr.org.cn, ChiCTR1800018714).

**Conclusion:** The use of absolute alcohol to embolize the middle meningeal artery could be used as an attempt to treat acute epidural hematoma and chronic subdural hematoma.

## Introduction

Traumatic brain injury is a common neurosurgical condition, and it often causes traumatic intracranial hemorrhage (TICH) (1). The TICH affects over 10 million people annually leading to either mortality or hospitalization (2). Subdural hemorrhage, epidural hemorrhage and intraparenchymal hemorrhage make up most TICHs (3). Patients with TICH frequently undergo neurologic deterioration due to an expanding hematoma. Even with surgical intervention, such patients experienced poor long-term outcomes (4).

Acute epidural hematoma (AEDH) is one of the most serious TICH and is often accompanied by sudden neurological symptoms (5). The most common cause is trauma leading to rupture of the middle meningeal artery (MMA), and progression is relatively rapid, sometimes requiring emergency surgery (6). Chronic subdural hematoma (CSDH) is a frequent TICH with incidence rates varying from 5.3 to 13.5 cases per 100,000 persons/year in the general population with a higher incidence among older adults (7). Surgical treatment is the first choice for TICH and consists of surgical evacuation using burr-holes or craniotomy (6, 8, 9). However, several studies reported a rate of re-expansion of acute small epidural hematomas after conservative treatment of 23 to 65% (10, 11), and some studies report a postoperative recurrence rate for CSDH of 0.35 to 23% or higher (12, 13). Furthermore, previous studies have found that there was a link between the expansion of AEDHs and the recurrence of CSDHs with the MMA. In order to prevent the re-expansion and recurrence of hematomas, they tried to use a minimally invasive method, MMA embolization in combination with burr-hole drainage (14–16). Currently, many materials were reported to achieve MMA embolization, and the embolization materials could be solid (polyvinyl alcohol particles, coils, and gelatin sponges) or liquid (n-butyl-2-cyanoacrylate) (17, 18). Absolute alcohol is the liquid sclerosing agent most often used because of its low cost, wide availability, and ease of use. It can destroy endothelial cells, and induce thrombosis by denaturing blood proteins, denuding the vascular wall of endothelial cells and precipitating their protoplasm (19–22). Alcohol has been widely-used in endovascular therapy to treat arteriovenous malformations, hemangioma, arteriovenous fistula, and other diseases, and is a popular material because of its low cost and efficacy (16, 22).

Although some studies report MMA embolization to treat intracranial hematoma, none, to our knowledge, have reported using absolute alcohol for this treatment. In this study, we report the successful use of MMA embolization with absolute alcohol to treat intracranial hematoma in 12 patients: five patients with AEDH and seven patients with CSDH.

## Materials and Methods

### Patient Selection

We retrospectively analyzed data for all patients with intracranial hematomas treated at our center between June 2018 and October 2019. Inclusion criteria of patients were: [1] all patients were clearly diagnosed as AEDH or CSDH in brain CT; [2] all patients' midline shift of the brain CT was <10 mm; [3] absolute alcohol was used in the MMA embolization. We excluded patients who underwent MMA embolization with other materials or new onset of dangerous anastomosis during intraoperative cerebral angiography and other underlying conditions (vascular lesions, brain tumor, arachnoid cyst, or previous craniotomy).

In the retrospective arm of the study, using the clinical data warehouse system of our institution, we reviewed 68 patients with AEDH or CSDH who were given a diagnosis, and 56 patients were excluded: 36 with AEDH or CSDH used other materials for MMA embolization; six with space occupying lesion (arachnoid cyst, intracranial tumor, et al.); five with urokinase injection; four with after craniotomy; three with vascular lesions (arteriovenous malformation, moyamoya disease, et al.), and two with dangerous anastomosis. Finally, according to our general neurosurgical diagnosis and treatment procedures, 12 patients were enrolled. A standard operative protocol was followed in all patients. We recorded patients' demographic information, clinical presentation, procedural methods, computed tomographic (CT) characteristics, angiographic changes, management, and outcomes. This study was carried out in accordance with the recommendations of the neurosurgical diagnosis and treatment guidelines, the ethic committee of Binzhou medical university hospital. The protocol was approved by our institutional ethics committee (No. 2018065). All subjects gave written informed consent in accordance with the Declaration of Helsinki.

### MMA Embolization

We performed MMA embolization under local anesthesia. First, the femoral artery was punctured using Seldinger's technique, and passage through the femoral artery was obtained using a standard 6-French sheath. This procedure was followed by external carotid arterial angiography, placing an Excelsior^®^ SL-10 microcatheter (Target Therapeutics/Boston Scientific, Fremont, CA, USA) under fluoroscopic guidance and placing a Traxcess^®^ 14 microguidewire (MicroVention, Tustin, CA, USA) into the trunk of middle meningeal artery followed by slow injection of absolute alcohol through the microcatheter for embolization (23, 24). Based on previous reports and our experience using absolute alcohol in our institution (25, 26), we mixed alcohol and contrast agent in an 8:2 ratios and used a dose of 0.03 ml/kg mixture for embolization, which we injected slowly. After injection, we removed the syringe and evaluated the extent of embolization. Also after each alcohol injection, and before removing the syringe, we waited for ~5 min to allow for complete action of the alcohol. Next, we removed the guiding catheter and the femoral arterial sheath and placed an occlusion in the femoral arterial puncture site. Generally, we treated each patient's hematoma condition in combination with burr-hole drainage to relieve their symptoms, improve prognosis, and reduce the recurrence rate or re-expansion rate. Postoperatively, all patients were transferred to the neurosurgical intensive care unit or ward for observation.

### Follow-Up

Hematomas in all patients were diagnosed or identified by CT scan on admission, and imaging was performed once per week or according to changes of the clinical manifestations in individual patients during hospitalization. All patients were discharged once their symptoms improved. A monthly repeat brain CT was recommended, and we followed all patients for 4–6 months after discharge. We readmitted patients if symptoms reappeared or worsened.

## Results

Table 1 shows the summary of the 12 cases. Ten patients were men, two were women, and their mean age was 54.2 years (range, 18–78 years). Ten patients had a history of head trauma. The Glasgow Coma Scale (GCS) on admission was higher than 13 in all patients. Among all patients, the most common clinical symptoms were headache, dizziness, drowsiness, vomiting, hemiparesis. All imaging data were confirmed preoperatively. All patients underwent absolute alcohol embolization of the middle meningeal artery, in combination with burr-hole drainage.

**Table 1:** Demographic data, clinical and imaging findings of the patients.

**Characteristics**		**Acute epidural hematoma (*N* = 5)**	**Chronic subdural hematoma (*N* = 7)**
Age	≤30	1	1
	30<, ≤60	3	2
	>60	1	4
clinical symptoms^a^	Headache	2	5
	Dizziness	2	2
	Drowsiness	1	1
	Vomiting	2	1
	hemiparesis	1	2
GCS^b^ score	14	3	4
	15	2	3
The size of hematoma (ml)	10<, ≤ 15	2	0
	15<, ≤20	3	1
	20<, ≤25	0	2
	25<, ≤30	0	4
Hematoma side	Left	2	4
	Right	3	3

a *Clinical symptoms data was a summary of symptoms rather than the number of patients*;

b *GCS: Glasgow Coma Scale*.

In all patients, embolization was successful, without complications, and bleeding was stopped immediately. There was no evidence of recurrent bleeding after the follow-up brain CT, and the hematoma cavity gradually decreased in size, thereafter. CSDHs may recur and AEDHs may expand after follow-up. However, these conditions were not identified during the follow-up period.

### Representative Cases' Clinical Data

#### Patient 3

**Figure 1:**
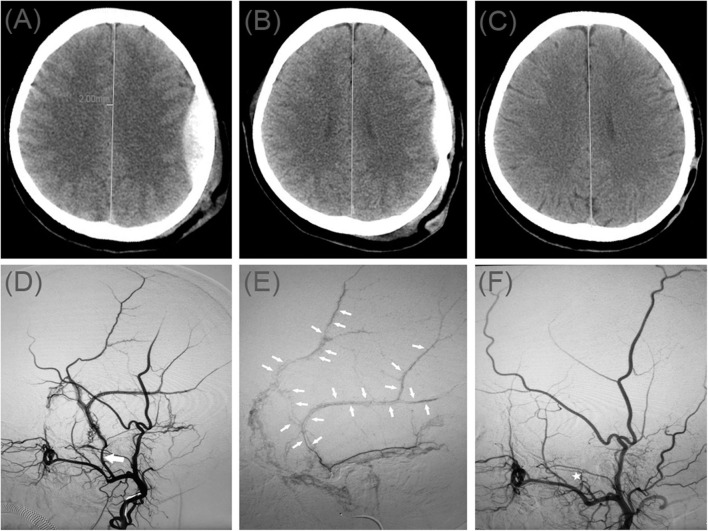
**(A)** Brain computed tomographic hematoma images for patient 1 before treatment (midline shift: 2.00 mm). **(B)** Brain CT was repeated on postoperative day 11. **(C)** Follow-up brain CT was performed at 88 days. **(D)** Pre-operative DSA presented abnormal MMA (Large arrow). **(E)** Intra-operative DSA showed contrast agent extravasation or arteriovenous fistula (Small arrow). **(F)** Postoperative DSA found that the MMA disappeared (Asterisk). DSA, digital subtraction angiographic.

A 50-year-old women injured in a traffic accident and was admitted to the hospital. His Glasgow Coma Scale score was 14, and clinical manifestations were mainly headache and dizziness. A CT scan disclosed a small EDH on the left side (Figure 1A). His symptoms changed on subsequent examinations, and a new symptom of vomiting appeared. In order to relieve the symptoms and prevent the enlargement of the hematoma, we took surgical treatment. The interval time between the injury and surgery was <2 h. First, he underwent MMA embolization. And subsequent burr-hole drainage was taken under local anesthesia. We performed cerebral angiography pre-, intra-, and postoperatively for MMA embolization (Figures 1D–F). His symptoms improved, and there were no signs of re-expansion or recurrence of the hematoma on CT brain scan before discharge. The hematoma cavity gradually decreased in size, thereafter (Figures 1B,C).

#### Patient 4

**Figure 2:**
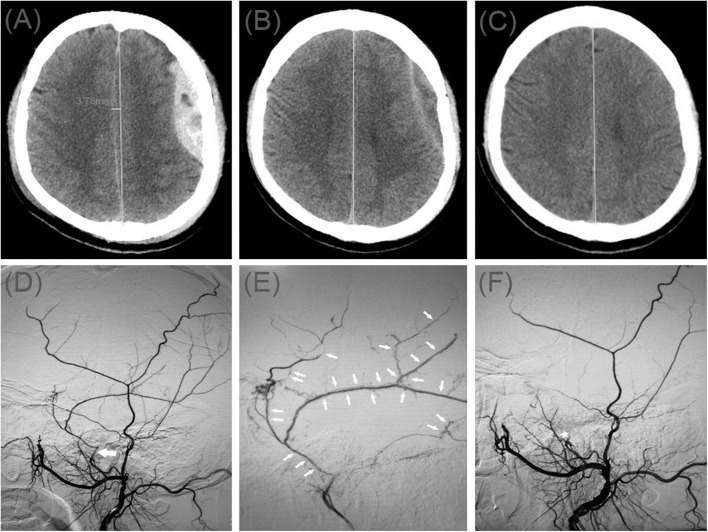
**(A)** Brain computed tomographic hematoma images for patient 2 before treatment (midline shift: 3.78 mm). **(B)** Brain CT was repeated on postoperative day 10. **(C)** Follow-up brain CT was performed at 67 days. **(D)** Pre-operative DSA presented abnormal MMA (Large arrow). **(E)** Intra-operative DSA showed contrast agent extravasation or arteriovenous fistula (Small arrow). **(F)** Postoperative DSA found that the MMA disappeared (Asterisk).

A 49-year-old man injured in an accidental fall and was admitted to the emergency room without neurological abnormalities. His Glasgow Coma Scale score was 14. A CT scan disclosed a AEDH on the left side (Figure 2A). On a subsequent neurological examination, his score on the Glasgow Coma Scale was 15. However, the headache symptoms of the patients were more obvious. In order to relieve the symptoms and prevent the enlargement of the hematoma, we took surgical treatment. The interval time between the injury and surgery was <2 h. First, he underwent MMA embolization. And subsequent burr-hole drainage was taken under local anesthesia. We performed cerebral angiography pre-, intra-, and postoperatively for MMA embolization (Figures 2D–F). His symptoms improved, and there were no signs of re-expansion or recurrence of the hematoma on CT brain scan before discharge. The hematoma cavity gradually decreased in size, thereafter (Figures 2B,C).

#### Patient 7

**Figure 3:**
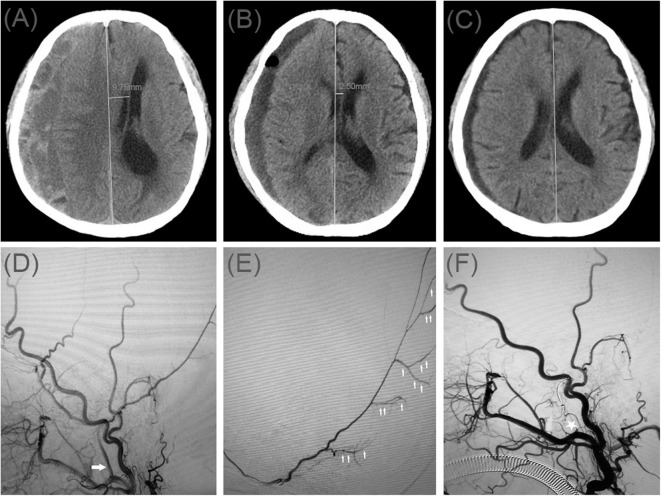
**(A)** Brain computed tomographic hematoma images for patient 3 before treatment (midline shift: 9.75 mm). **(B)** Brain CT was repeated on postoperative day 12 (midline shift: 2.50 mm). **(C)** Follow-up brain CT was performed at 58 days. **(D)** Pre-operative DSA presented abnormal MMA (Large arrow). **(E)** Intra-operative DSA showed contrast agent extravasation or cotton wool-like staining (Small arrow). **(F)** Postoperative DSA found that the MMA disappeared (Asterisk).

A 70-year-old man was admitted to our hospital for weakness in his left limb. He had a head injury 3 months ago. His Glasgow Coma Scale score was 14. A CT scan disclosed multiple site SDH on the right side (Figure 3A). His GCS score improved on subsequent examination, but his left lower limb was gradually weak, which presented only four levels of muscle strength. In order to relieve symptoms and recurrence of hematoma, we took surgical treatment. First, he underwent MMA embolization and burr-hole drainage under local anesthesia. We performed cerebral angiography pre-, intra-, and postoperatively for MMA embolization (Figures 3D–F). No imaging of the middle meningeal artery was observed in postoperative angiography, and postoperatively, his symptoms improved. There was no evidence of rebleeding after repeat brain CT before discharge, and the hematoma cavity gradually decreased in size, thereafter (Figures 3B,C).

#### Patient 9

**Figure 4:**
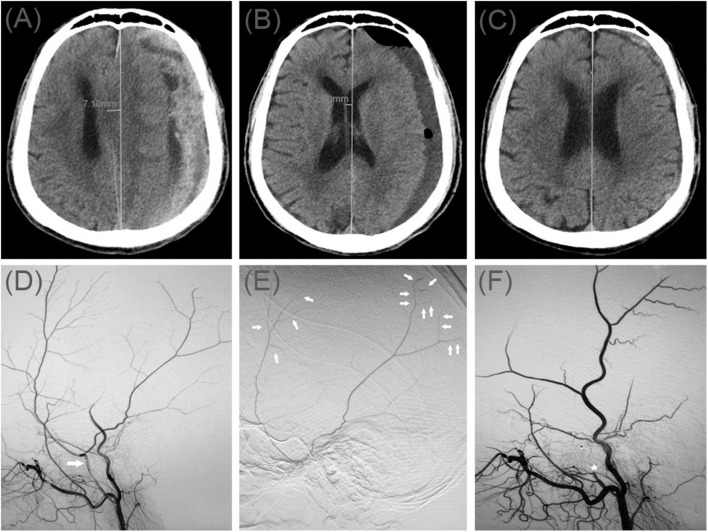
**(A)** Brain computed tomographic hematoma images for patient 4 before treatment (midline shift: 7.10 mm). **(B)** Brain CT was repeated on postoperative day 14 (midline shift: 2.00 mm). **(C)** Follow-up brain CT was performed at 72 days. **(D)** Pre-operative DSA presented abnormal MMA (Large arrow). **(E)** Intra-operative DSA showed contrast agent extravasation or cotton wool-like staining (Small arrow). **(F)** Postoperative DSA found that the MMA disappeared (Asterisk).

A 78-year-old man was admitted to our hospital with a duration of sudden-onset headache. He had a head injury 2 months ago. His Glasgow Coma Scale score was 14. A CT scan disclosed multiple site SDH on the left side (Figure 4A). On a subsequent neurological examination, his symptoms was more serious in headache and a new symptom of vomiting appeared. First, he underwent MMA embolization and burr-hole drainage under local anesthesia. We performed cerebral angiography pre-, intra-, and postoperatively for MMA embolization (Figures 4D–F). No imaging of the middle meningeal artery was observed during postoperative angiography, and postoperatively, his symptoms improved. There was no evidence of recurrent bleeding after repeat brain CT before discharge, and the hematoma cavity gradually decreased in size, thereafter (Figures 4B,C).

## Discussion

In our study, we treated 12 patients with TICH who underwent minimally-invasive treatment with absolute alcohol embolization of the MMA. According to their symptoms and imaging findings, all patients underwent MMA embolization in combination with burr-hole drainage under local anesthesia. The purpose of embolization was to treat the bleeding source and reduce the re-expansion or recurrence of hematoma, and the purpose of burr-hole drainage was to alleviate patients' symptoms. In our study, all patients' symptoms alleviated postoperatively, and patients were discharged after symptom improvement. Based on brain CT findings, no patients experienced hematoma recurrence after discharge, and the hematoma cavity decreased significantly in size over time.

### Mechanisms for the Development of AEDH and CSDH, and Related Digital Subtraction Angiography (DSA) Findings

AEDH is a rapid-onset and severe traumatic brain injury (5). Arterial bleeding is responsible for 85% hematomas in AEDHs. Among them, the middle meningeal artery (MMA) is the most common source of EDHs (10, 27). To begin with, small EDHs are usually treated conservatively. Nevertheless, in some large samples of EDHs, it was found that the rate of re-expansion of hematoma after conservative treatment reached 5.5~65% (9, 28). Sakai et al. showed that 65% of small EDHs re-expansion in the first 24 h after trauma (29). A prospective study evaluating 22 patients who had small EDHs showed that 32% of the patients underwent surgical evacuation because of late enlargement (30). Furthermore, most studies of EDHs suggest that EDHs are related to tearing or damage to the MMA, seen as contrast agent extravasation, pseudoaneurysm, or arteriovenous fistula in DSA (28, 31). However, the best treatment option for EDH in larger or occipital and non-middle meningeal arterial blood supply areas remains surgery (32).

CSDH is being diagnosed more commonly in neurosurgical practice, and burr-hole drainage is the gold standard for patients with space-occupying CSDH (12). One hypothesis for the cause of CSDH is tearing of one or more bridging veins, which causes bleeding into the dural border cell layer and leads to CSDH enlargement (7, 8). However, evidence currently indicates that the responsible blood vessels for CSDH may arise from the MMA. Takizawa et al. observed that the MMA was enlarged with CSDH (33). Several studies have shown that selective MMA angiography revealed diffuse dilatation of the MMA and visualization of scattered abnormal vascular networks reflecting macrocapillaries, a sinusoidal channel layer, and giant capillaries in the outer membrane (13, 34). Additionally, a “cotton wool-like staining” appearance of the distal MMA vasculature and “wispiness” (or the neovasculature) of the distal MMA branches may be observed in DSA (35).

### Treatment for AEDH and CSDH

MMA embolization has been reported for the treatment of EDH (11, 36). The first published series of the endovascular treatment of AEDH was described by Suzuki et al. (6), and the authors reported successful embolization in nine patients with EDHs and associated lesions. Zhang et al. reported a novel minimally-invasive method of endovascular embolization under local anesthesia to treat AEDH, which is especially useful for older patients or patients unable to undergo general anesthesia (10). Furthermore, to date, the use of urokinase with clot aspiration has emerged as the most promising surgical modality for intracerebral hemorrhage and intra-ventricular hemorrhage (5, 37, 38). According to this fact, some researchers resorted to urokinase to facilitate the liquifaction of lots for aspiration of the AEDH. However, early application of urokinase may have a certain risk of bleeding, which is generally used after operation (10, 38, 39). This purpose can effectively dissolve the hematoma without increasing the risk of long-term bleeding. The urokinase (20 ku resolved in 3-mL saline, twice a day) was injected into the hematoma cyst by drainage tube to lyse hematoma on the first postoperative day. The reason why our patients did not use urokinase was that our patients had a relatively small amount of hematoma, MMA embolization and burr-hole drainage without urokinase was enough for preventing hematoma expansion and removing the occupying effect of hematoma. Peres et al. and Ross et al. demonstrated that MMA embolization was highly effective and safe to achieve size stabilization in non-surgically-treated AEDH (28, 40).

Treatment for CSDH should address the capillary feeders of the hematomas that originate from the MMA. Hashimoto et al. found that MMA embolization was effective for refractory CSDH or CSDH patients with a risk of recurrence, and this method is considered effective to stop hematoma enlargement and promote resolution (41). Link et al. and Kim et al. reported that MMA embolization was a minimally-invasive and low-risk initial alternative to surgery for symptomatic CSDH when clinically appropriate (13, 17).

According to the above related reports, our research has tried to use absolute alcohol to embolize the middle meningeal artery to treat AEDH or CSDH, and observed that absolute alcohol was feasible as an embolization agent for middle meningeal artery embolization.

### Important Considerations in MMA Embolization

MMA embolization is a rapid technique, but a complete angiographic examination should be performed before embolization. To begin with, Studies have shown that anastomotic branches can be present between the external- and the internal carotid arteries, and inappropriate embolization of these anastomotic branches can cause serious complications; sometimes the central retinal artery originates from the MMA (10, 30). Intraoperatively, surgeons should check for an anastomosis between the MMA and the ophthalmic artery. If a dangerous anastomosis is observed, embolization injection should be performed carefully. Studies have reported the presence of extracranial–intracranial anastomoses of the external carotid artery with an average diameter of > 100 um (10, 28, 42). Generally, previous studies used embolization materials with a diameter much larger than the diameter of the anastomoses; therefore, no complications were seen related to the embolization material entering the intracranial blood vessels through the anastomosis and leading to cerebral infarction or cranial nerve dysfunction. In our study, we excluded patients with a dangerous anastomosis, thus we did not observe a complication due to inappropriate embolization during our follow-up. Furthermore, there was currently no agreement on the site of MMA embolism: trunk or distal (11, 17, 18). Link et al. suggested that during embolization, physicians should proceed carefully to avoid inappropriately embolizing the petrosal branch to avoid facial nerve dysfunction (35). However, other studies showed that the petrosal branch was not the only blood supply to the facial nerve (43). This may be the reason why we embolized the trunk of MMA without new symptoms.

### Use of Alcohol in Endovascular Interventional Therapy

Alcohol has been widely-used in endovascular therapy because of its low cost and ease of access. Alcohol is effective in the intravascular treatment of vascular malformations, dysfunctions, and hematomas (14, 22). Alcohol may act by promoting coagulation of proteins in the blood or lymphatic fluid and destroying the cellular lining of the abnormal vessel (26). Singh et al. found that absolute alcohol embolization was safe and effective to treat vertebral hemangiomas with severe spinal cord compression (24). Additionally, Chen et al. demonstrated that using absolute alcohol for percutaneous embolization to treat venous malformations was safe and effective (15). The volume of alcohol injected is based on the size and location of the malformation and the patient's weight (44), and injection is stopped when resistance to manual injection increases or when alcohol is visualized exiting the lesion in the venous drainage (45). The maximum recommended and safe dose of alcohol is 1 ml/kg body weight and can be used with proper precautions (21, 44, 45). Based on previous reports and our experience, we explored an appropriate amount of alcohol for embolization of the blood vessels responsible for intracranial hematoma.

The results of this study are promising, but our conclusions are limited. The number of patients in this study was small, and the study lacked a control group. We also did not compare our procedure with previous treatments or embolization with other embolic materials. We also had no standard way to measure outcomes; therefore, there may be limitations related to our measurements. Prospective randomized case-control studies are needed to evaluate the efficacy of absolute alcohol embolization, and we expect that not all patients will be treated successfully using this technique. Despite the limitations, embolization provided a possible new method of MMA embolization in younger patients or those intolerant to general anesthesia. Absolute alcohol MMA embolization could also become a new, low-cost treatment for refractory problems. Our approach could make minimally-invasive MMA embolization more available.

## Conclusions

The effect and prognosis in patients receiving absolute alcohol MMA embolization in our study were similar to results in studies using other embolic materials. Because of its low cost and ease of access, it may be used as a recommended MMA embolization material in the future when treating AEDH and CSDH.

## Data Availability Statement

The datasets generated for this study are available on request to the corresponding author.

## Ethics Statement

Written informed consent was obtained from the individuals for the publication of any potentially identifiable images or data included in this article.

## Author Contributions

GF drafted the manuscript. CW and CX completed the operation. HW, JD, and YL performed the data collection and data analysis. CW and ZL participated in the design of this study and helped to check the manuscript. All authors read and approved the final manuscript. All authors contributed to the article and approved the submitted version.

## Conflict of Interest

The authors declare that the research was conducted in the absence of any commercial or financial relationships that could be construed as a potential conflict of interest.
